# Slipped upper femoral epiphysis with hemophilia A

**DOI:** 10.4103/0019-5413.33694

**Published:** 2007

**Authors:** Deepa Iyer, Richard Brueton

**Affiliations:** Department of Orthopedics, Royal Free Hospital, Pond Street, London NW3 2QG, UK

**Keywords:** Hemophilia A, slipped upper femoral epiphysis

## Abstract

A 13-year-old boy who had hemophilia A was reported with pain in the left thigh and hip on walking. He had no history of trauma. Severe hemophilia A is diagnosed with a Factor VIII level of <1 iu/dl. The presumptive diagnosis was that of a spontaneous bleed into the hip joint. Factor VIII mutational analysis revealed a C to G substitution at nucleotide 6683 which results in a cystine change at codon 2194. However, the symptoms persisted and an X-ray demonstrated the presence of an acute on chronic slip of the upper femoral epiphysis. The patient was transferred to the center treating his hemophilia where the hip was pinned *in situ* under cover with Factor VIII. This case demonstrates the need to be aware of a possible traumatic diagnosis of hip pain in a hemophiliac child with a longstanding history of spontaneous bleeding into joints.

The occurrence of a slipped upper femoral epiphysis in a child with hemophilia A has not been reported. We present such a case and discuss the difficulty in reaching a diagnosis.

## CASE REPORT

**Figure 1:**
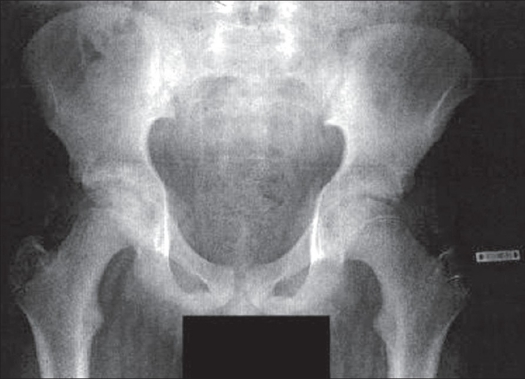
X-ray (Antero posterior) of pelvis: Pre operative. imaging looks normal, with no evidence of SUFE, which can mislead diagnosis

A 13-year-old boy was admitted to his local hospital, with a six-week history of increasing pain in his left lateral thigh on walking. There was no definite history of trauma. Since the child suffered from severe haemophilia A, the mother suspected a bleed into the left hip. A diagnosis of bursitis had been made but the pain persisted, being worse on activity. After consulting his GP, he was referred to the local accident and emergency department where X-rays of the hips as certained the diagnosis of slipped upper femoral epiphysis [Figure 1]. He was referred for further management to the center where his hemophilia was being managed.

This child had been diagnosed with hemophilia A at the age four months and was on regular treatment with recombinant factor VIII (Kogenate Bayer) 2500 IU - three times/week. Despite this treatment, he still had -two to three breakthrough bleeds each month, in either the ankles or the knees. One year back he had an osteochondral fracture of the medial patellar facet (diagnosed by MRI) that was treated by arthroscopic removal of fragment (2.5 × 1 cm). On examination, he could flex his left hip up to 90° that lay in 30° of external rotation. He had no pain on the movement of the hip.

**Figure 2:**
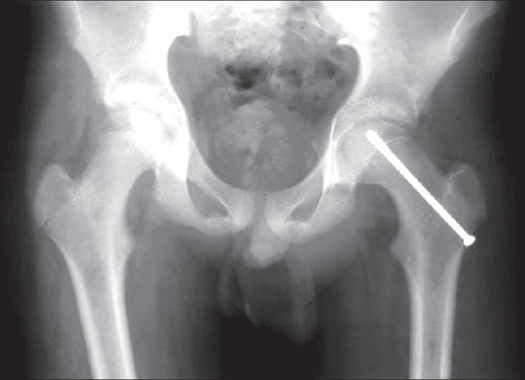
X-ray (Antero - posterior): Post operative with a cannulated screw

An antero-posterior X-ray of the pelvis and lateral X-ray of the hip, showed a slip of the left upper femoral epiphysis. There was no evidence of avascular necrosis. He underwent in situ fixation of a left slipped upper femoral epiphysis, under general anesthesia by means of one cancellous screw [Figure 2]. Postoperative recovery was uneventful and he mobilized well with crutches. He has been maintained on regular follow-up and has had excellent recovery ever since.

## DISCUSSION

Slipped upper femoral epiphysis (SUFE) is a Salter-Harris Type 1 fracture through the proximal femoral physis.^1^ The fracture occurs at the hypertrophic zone of the physeal cartilage and may compromise the blood supply to the femoral head leading to avascular necrosis. Manipulation is therefore contraindicated as this may further compromise the blood supply and pinning of the hip is carried out in situ. Stabilizing the hip by the insertion of a screw to cross the physis, reduces further damage to the penetrating vessels. An upper femoral osteotomy may be indicated later if there is significant residual external rotation and extension at the hip.

Hemophilia A is an X-linked, recessively inherited bleeding disorder which results from deficiency of procoagulant Factor VIII.^2^,^3^ Affected males suffer from joint and muscle bleeds and easy bruising, the severity of which is closely correlated with the level of activity of coagulation factor VIII (FVIII:C) in their blood. Factor VIII deficiency, dysfunctional F (VIII) or F (VIII) inhibitors lead to the disruption of the coagulation cascade, resulting in spontaneous hemorrhage and/or excessive hemorrhage in response to trauma. Recurrent hemarthroses lead to specific changes in both the synovium and cartilage, which finally result in the destruction of the joint.

Children with severe hemophilia A often begin to show increased bruising around the age of one year.^4^,^5^ It may present earlier, if the child undergoes surgery or after minor trauma, including immunizations. The major problem in severe Hemophilia A is painful bleeding into joints, mainly the knees, ankles and elbows. The bleeds may occur spontaneously. If not treated promptly, the bleeds may result in permanent arthritis and disability. Slipped upper femoral epiphysis is an uncommon condition and the presence of underlying hemophilia may distract the physician from reaching the diagnosis. In a child with a previous history of repeated joint pains, the clinician must be alert to a new pathology presenting with similar symptoms. Slip of the upper femoral epiphysis is as likely to occur in children with hemophilia as in the general population. The physician must therefore maintain a high index of suspicion in this group of patients concerning the differential diagnosis.

Although bleeding into joints is the most common cause of joint pain in children with hemophilia, alternative diagnoses must be considered in children presenting with joint pain. This patient was initially thought to have had a spontaneous bleed into the hip joint; the correct diagnosis of an acute on chronic slip of the upper femoral epiphysis was reached and the patient treated accordingly.
